# Potentially inappropriate prescriptions for elderly people taking antidepressant: comparative tools

**DOI:** 10.1186/s12877-017-0674-2

**Published:** 2017-12-02

**Authors:** Izabela Fulone, Luciane Cruz Lopes

**Affiliations:** Pharmaceutical Sciences Post graduate Course, University of Sorocaba, UNISO, Rodovia Raposo Tavares, KM 92,5, Sorocaba, São Paulo ZIP Code 18023-000 Brazil

**Keywords:** Elderly, Psychotropic drugs, Antidepressants, Screening tool, Inappropriate medications, Potentially

## Abstract

**Background:**

The use of psychotropic drugs by elderly people is widely spread around the world, given that prevalence of inappropriate medication is frequent. Strictly speaking, in Brazil, the vulnerable population of elderly people is more likely to use Potentially Inappropriate Psychotropic (PIP) due to the impact of social-economic characteristics, to the Brazilian Public Health System, and to the lack of patient monitoring. However, neither the use pattern nor the prevalence rate of PIP have been studied in Brazil so far. The objectives of this study were to determine the prevalence of PIP in elderly outpatients taking antidepressants, and to compare the performance of two different tools (Beers, STOPP).

**Methods:**

This cross-sectional study involved all the aged outpatients (≥ 60 years of age) taking antidepressants attended by the public health system in a city of the State of São Paulo, Brazil. Data were obtained from a pharmacy database and medical records. All psychotropic drugs evaluated included: antidepressants, antipsychotics, anti-epileptics and benzodiazepines. STOPP and Beers criteria were applied to detect PIP.

**Results:**

One thousand one hundred forty prescriptions from 174 outpatients were subjected to two different screening tools. The average patient age was 67 (interquartile range 63–74) and the median number of drugs used was 3.0 (interquartile 2–4) per patient. The overall prevalence of PIP was 121 (69.5%). The levels of PIP observed according to tools were 39.6% (STOPP) and 29.9% (Beers).The long-term use of benzodiazepines was the most common PIP recognized, and the one which contributed more significantly to higher levels of PIP than other medications.

**Conclusions:**

The prevalence of PIP was high among the elderly. STOPP criteria identified more PIP than Beers criteria. Knowledge of PIP prevalence should gear efforts to reduce the level of inappropriate prescriptions and may provide the need for developing national criteria.

## Background

Depression is among the most common psychiatric disorders in geriatric patients, and occurs even more frequently than dementia [1]. It is underdiagnosed and undertreated [2]. Depressed elderly people are more likely to die than the non-depressed elderly ones, and few receive appropriate therapeutic interventions [3].

Factors significantly associated with depression in the elderly include: chronic health problems, functional impairment, cognitive impairment, lack of close social contacts, and previous history of depression [2].

Approximately 50% of patients in this age group have important clinical improvement as a result of treatment with antidepressants [3]. On the other hand, they are also more prone to cardiovascular events, and suffer anticholinergic and cognitive decline related to various antidepressants [4]. Physical illness and handicap may affect the efficacy and tolerance to antidepressant treatment [3].

The high prevalence of potentially inappropriate medication (PIM) is associated with increased morbidity, mortality and decrease in quality of life. As the number of medications increase, patients become increasingly at risk of Adverse Drug Reactions. It is estimated that 30% of elderly people’s admissions in hospitals is due to problems related to drugs or to toxic effects of drugs [5].

Antidepressants, anxiolytics and antipsychotics are the most common potentially inappropriate psychotropic (PIP) [6]. Tricyclic antidepressants have manyknow contraindications such as dementia, cardiac conductive abnormalities, chronic constipation, prostatism or history of urinary retention [7]. So, tricyclic antidepressants should be avoided for being highly anticholinergic, sedating and causing orthostatic hypotension [8]. Selective serotonin reuptake inhibitors (SSRIs) should be used with caution, as they may exacerbate or cause syndrome of inappropriate antidiuretic hormone secretion or hyponatremia [8, 9]. Besides that, some drug-drug interactions involving antidepressants offer risks, i.e. tricyclic antidepressants and SSRIs, shows increased risk of falls [8].

Screening tools to detect PIM for the elderly have been developed in several countries. Such tools contribute to measure the quality of care in the elderly age group, so as to make rational clinical decision to mitigate problems related to drugs and adverse events [10]. The most common explicit criteria are STOPP (Screening Tool in Older Persons’ Prescriptions) and Beers.

STOPP criteria were formulated and validated in European countries and are based on the physiological systems, considering drug-drug and drug-disease interactions, dose and duration of treatment [7]. Beers criteria were originally proposed more than two decades ago and have been updated several times, most recently in 2015. These criteria were developed in the US context, and include: PIM and classes of medication to be avoided in elderly people; PIM with certain diseases and syndromes; medications to be used with caution in elderly people; selection of drugs that should be avoided or their dose adjustment based on the individual’s kidney function; and the selection of drug-drug interactions documented to be associated with harms in older adults [8].

Much has been published about the prevalence of psychotropic drug use, particularly antidepressant drugs, in elderly people around the world, but there are few studies in Brazil focusing over-prescription, misprescription, under-prescription and safety of these medications in this specific and vulnerable population [11, 12]. Besides that, a systematic review shows that drug utilization studies comparing data cross-nationally are scarce in Latin America and the validity of the data to ensure the comparability is hampered due the lack of available data on drug consumption from the public healthcare [13].

Many studies have documented changes in prescription over time, showing decreases in tricyclic antidepressants, first generation antipsychotic, and long-acting benzodiazepines, in accordance with guidelines from other countries (e.g. North America and Europe) [14–16]. Research conducted in Canada has shown an increase in the use of antidepressantsand a substantial shift in prescribing practices with the SSRIs and tricyclic antidepressants, in which the use of SSRIs has more than quadrupled and the use of tricyclic antidepressants has fallen by almost 50% in the 5 years’ observation study [14].

The use of long-acting benzodiazepines, another class of drugs that have always been prescribed together with antidepressant drugs, seems to have decreased and benzodiazepine-related drugs (called Z drugs) have shown higher probability of use [17]. Meanwhile, some studies developed in Brazilian settings [11, 18–20] involving this type of drugs, show that we have been using these drugs and we do not know the extension of use of prescribing tools to measure this problem.

Thus, prescription of inappropriate medication to the elderly population has become a public health problem worldwide, despite being a preventable problem. Considering the high percentage of aged people taking antidepressants, this study aimed to determine the prevalence of potentially inappropriate psychotropic among elderly people taking antidepressants according to STOPP criteria [7] and Beers criteria [8].

## Methods

### Design, settings and context

This cross-sectional study investigated all elderly outpatients (≥ 60 years of age) taking antidepressants, treated in the public health system of Porto Feliz, State of São Paulo, Brazil, as well as their respective prescriptions.

The study used the centralized database of the public pharmacy outlet for dispensing drugs under special control of the city and we collected information available from January 2008 to December 2009.

Porto Feliz has a population about 52,000 inhabitants and 16% of whom living in rural areas [21]. Its economy is based on agriculture and it has a high Human Development Index. Approximately 75% of its inhabitants are assisted by its public health service, which includes physician visits, diagnostic procedures, and access to medication.

Ethics approval for the present study was granted by the Ethics Committee for Clinical Research of the University of Sorocaba in April 2010, with protocol number 003/2010.

### Eligibility criteria and data collection

Firstly, the study identified all the elderly taking antidepressants in the pharmacy database. From the identified records in the pharmacy database, we located the medical records of each antidepressant user. Medical records of patients already dead as well as of those with insufficient information were excluded from the study.

The data from the dispensing system of the pharmacy and the medical records constituted a database that contained the following information: gender, age, marital status, presence of comorbidities, concurrent use of drugs with antidepressant, type of diagnoses, type of antidepressant and use of other psychotropic drugs (benzodiazepines, antipsychotics and anti-epileptics). All this information is linked with individual ID numbers in the public health system.

All the data from concurrent medicines during the antidepressant treatment were analysed. We considered as concurrent medicines all those prescribed in the same time of use as the antidepressant. This way, all the other drugs dispensed in the time of prescription of antidepressant for each aged person included in this study from January 2008 to December 2009 were cataloged.

The drugs were classified according to the Anatomical Therapeutic Chemical System (ATC) [22]. International Classification of Disease version 10 (ICD-10) codes were used to identify the medical diagnoses and comorbidity conditions [23].

To determine the use of inappropriate psychotropic drugs in elderly patients and the inappropriate prescriptions of psychotropic, STOPP [7] and Beers criteria were applied (version updated 2015) [8].

By the American Geriatrics Society 2015 Beers Criteria Update Expert Panel, we considered two lists to check the data from medical records: (i) list of PIM for older adults outside the palliative care and hospice setting, including medications to be avoided for many or most older adults; (ii) medications to be avoided for older adults with specific diseases or syndromes.

Continuous variables were described by means and standard deviations or median, minimum and maximum values as appropriate, whereas proportions described binary variables.

## Results

**Fig. 1:**
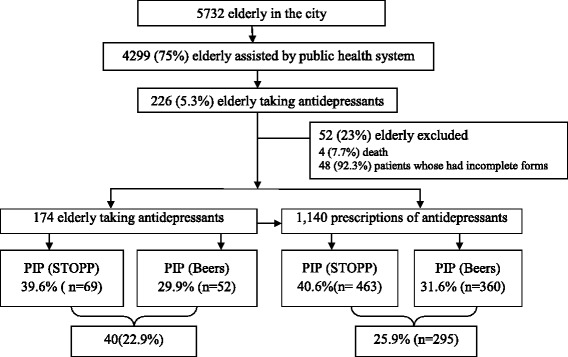
Presence of PIP in elderly people taking antidepressants

Out of 4299 elderly people assisted by public health system in this city, 226 (5.25%) were taking antidepressant during the study time. Excluding those who had died (*n* = 4) and others with incomplete data (*n* = 48), the eligible people from this study were 174 (4.04%). The total number of antidepressant prescriptions for all elderly people eligible was 1140. Considering both criteria (STOPP + Beers), we found PIP in 251 (25.9%) prescriptions to 40 (22.9%) patients, as shown in Fig. 1.

Table 1 shows characteristics of elderly people taking antidepressants included in this study versus results from PIP (Beers) and PIP (STOPP). Out of 174 elderly people,141 (81.1%) were female, with median age 67 (interquartile range 63–74) and the median number of drugs used was 3.0 (interquartile 2–4) per patient. In general, only 19 (10.9%) patients did not use concurrent drugs with the antidepressant and 155 (89.1%) used at least one or more concurrent drugs. Considering all concurrent drugs used with the antidepressants, we highlight the use of drugs that played role in the central nervous system, drugs that acted on the renin angiotensin system, diuretics and drugs used in diabetes. The most common types of diagnoses were major depression-F32 (41.4%) and anxiety-F41 (35.1%). Cardiovascular diseases (47.1) and diabetes (13.2%) were the most prevalent comorbidities.

**Table 1:** Characteristics of elderly people taking antidepressants and prevalence of PIP

Characteristics	TOTAL	PIP Stopp	PIP Beers	PIP Both
	*n* = 174 (100%)	*n* = 69 (%)	*n* = 52 (%)	*n* = 40 (%)
Age (years)				
*60–74*	135 (77.6)	56 (81.1)	43 (82.7)	34 (85.0)
*≥ 75*	39 (22.4)	13 (18.9)	9 (17.3)	6 (15.0)
Sex				
*Female*	141 (81.1)	58 (84.1)	45 (86.5)	34 (85.0)
*Male*	33 (18.9)	11 (15.9)	7 (13.5)	6 (15.0)
Concurrent drugs				
*None*	19 (10.9)	–	–	–
*1*	62 (35.6)	22 (31.9)	16 (30.8)	12 (30.0)
*≥ 2*	93 (53.4)	47 (68.1)	36 (69.2)	28 (70.0)
Comorbidities				
*None*	63 (36.2)	21 (30.4)	19 (36.5)	14 (35.0)
*1*	66 (37.9)	26 (37.7)	19 (36.5)	16 (40.0)
*2*	37 (21.3)	19 (27.5)	12 (23.1)	9 (22.5)
*3*	8 (4.6)	3 (4.3)	2 (3.8)	1 (2.5)
Comorbidities				
*Cardiovascular disease* ^*a*^	82 (47.1)	41 (59.4)	24 (46.1)	19 (47.5)
*Diabetes mellitus*	23 (13.2)	10 (1.4)	5 (9.6)	4 (10.0)
*Hypothyroidism*	12 (6.9)	3 (4.3)	3 (5.8)	3 (7.5)
Diagnostic for prescription of antidepressants^b^				
*Depression*	72 (41.4)	35 (50.7)	46 (88.5)	34 (85.0)
*Anxiety*	61 (35.1)	27 (39.1)	5 (9.6)	5 (12.5)
*Other* ^*c*^	56 (32.2)	18 (26.1)	8 (15.4)	8 (20.0)

^a^hypertension, arrhythmias, heart failure;

^b^patient can have more than one type of diagnosis;

^c^dissociative disorder, reaction to severe stress and adjustment disorders, migraine

Regarding only the number of psychotropic drugs which played role in the central nervous system, approximately 76 (43%) of the elderly used only antidepressants, 69 (39.6%) of them used at least on one type of psychotropic concomitant with antidepressant and 29 (16.7%) used more than two types of psychotropic drugs concomitant with the antidepressant. The psychotropic drugs most associated to the use of antidepressants were: benzodiazepines (45.4%), antipsychotics (15.5%), anti-epileptics (6.3%), Table 2.

**Table 2:** Type of antidepressants and concomitant use of other psychotropic drugs

Use of psychotropic drug	*n* = 174 (%)
Type of antidepressants	
Selective serotonin reuptake inhibitor^a^	95 (54.6)
Tricyclic antidepressants^b^	73 (41.9)
Other antidepressants^c^	6 (3.4)
Concomitant use of benzodiazepines	79 (45.4)
Benzodiazepines with short-intermediate half-life^d^	46 (58.2)
Long half-life benzodiazepines^e^	33 (41.7)
Concomitant use of antipsychotics-N05A^f^	27(15.5)
Concomitant use of antiepileptics-N03^g^	11 (6.3)

^a^fluoxetine, citalopram, sertraline

^b^amitriptyline, imipramine and clomipramine

^c^bupropion

^d^alprazolam, bromazepam, clonazepam, lorazepam, nitrazepam

^e^diazepam

^f^fluphenazine, chlorpromazine, haloperidol, levomepromazine, thioridazine, risperidone

^g^carbamazepine, phenytoin, phenobarbital

The number of PIP observed according to the two tools was 39.6% (STOPP) and 29.9% (Beers). STOPP identified 6 types of PIP affecting 69 (39.6%) patients in 463 (40.6%) inappropriate prescriptions. Beers criteria identified 4 types of PIP affecting 52 (29.9%) patients in 360 (31.6%) inappropriate prescriptions. The most common inappropriate psychotropic drug recognized in both tools was the long-term use of benzodiazepines, as shown in Table 3.

**Table 3:** PIP and PIM by STOPP and Beers criteria in elderly taking antidepressants

Condition/	Drug	PIM number of patients	PIP in prescriptions
STOPP criteria		*n* = 69 (%)	*n* = 463 (%)
Dementia	tricyclic antidepressants	1(1.4)	2 (0.4)
Glaucoma	tricyclic antidepressants	1 (1.4)	5 (1.1)
Cardiac conductive abnormalities	tricyclic antidepressants	5 (7.2)	32 (6.9)
Use of calcium channel blocker	tricyclic antidepressants	3 (4.3)	5 (1.1)
Long-term (> 1 month)	long-acting benzodiazepines and benzodiazepines with long-acting metabolites ^a^	54 (78.3)	397 (85.7)
Long-term (> 1 month)	neuroleptics as long-term hypnotics	15(21.7)	96(20.7)
Beers criteria		*n* = 60 (%)	*n* = 403 (%)
Arrhythmias	tricyclic antidepressants	4(6.6)	32(7.9)
Depression	long-term benzodiazepines use	41(68.3)	287(71.2)
Depression	methyldopa	1(1.6)	5 (1.2)
Concomitant use ≥3 CNS-active drugs^b^	tricyclic antidepressant + SSRI + benzodiazepine + antipsychotic	1 (1.6)	3 (0.7)
	tricyclic antidepressant + SSRI + antipsychotic	2(3.3)	4 (1.0)
	antidepressant + benzodiazepine + antipsychotic	10 (16.6)	64 (15.9)
	antidepressant + benzodiazepine + two antipsychotics	1 (1.6)	8 (1.9)

*SSRI* selective serotonin reuptake inhibitor

^a^clonazepam; diazepam;

^b^ CNS drugs = central nervous system active drugs: antipsychotics; benzodiazepines; tricyclic antidepressants; SSRIs;

Considering the use of the type of medication “independent of diagnosis or conditions”, the level of PIP by Beers criteria was higher 864 (75.8%). The main PIP’s found in 864 prescriptions were: amitriptyline 276 (31.9%), diazepam 263 (30.4%), imipramine 217 (25.1%), clonazepam 178 (20.6%) and haloperidol 91 (10.5%), as shown in Table 4.

**Table 4:** Potentially inappropriate psychotropic drugs (PIP) and Potentially inappropriate medicines (PIM) for elderly people according to Beers criteria “independent of diagnosis or conditions”

Drug	PIM number of patients *n* = 132 (100%)	PIP prescriptions *n* = 864 (100%)
Tricyclic antidepressants		
*amitriptyline*	41 (31.1)	276 (31.9)
*clomipramine*	8 (6.1)	52 (6.1)
Benzodiazepines		
*alprazolam*	1 (0.7)	8 (0.9)
*lorazepam*	3 (2.3)	34 (3.9)
*clonazepam*	26 (19.7)	178 (20.6)
*diazepam*	36 (27.3)	263 (30.4)
Antipsychotics, first and second generation		
*haloperidol*	12(9.1)	91 (10.5)
*chlorpromazine*	7 (5.3)	36 (4.1)
*thioridazine*	2 (1.5)	7 (0.8)
*risperidone*	1 (0.7)	7 (0.8)
Barbiturates (antiepileptics)		
*phenobarbital*	2 (1.5)	17 (19.7)

## Discussion

### Main findings

The data revealed high prevalence of PIP in the elderly taking antidepressants. Considering diagnoses and condition, STOPP identified more PIP than Beers criteria. The most common PIP recognized in both tools was the long-term use of benzodiazepines.

When we considered Beers criteria “independent of diagnoses or conditions”, the results of PIP were higher, mainly comprising the use of some tricyclic antidepressants (amitriptyline, clomipramine and imipramine) and benzodiazepines (clonazepam, diazepam, alprazolam, lorazepam).

### Comparison with other studies

In the latest years, the consumption of antidepressants by the elderly has increased significantly and potentially inappropriate prescription is frequent, mainly in community-dwelling older adults with depressive symptoms [24]. The older adults are 7 to18 times more likely to use psychotropic drugs than middle-aged adults [25].

In our study, more than 50% of the elderly used concurrent psychotropic drug with the antidepressant. The most common association was antidepressant plus benzodiazepine or antidepressant plus antipsychotic. This trend was also observed in the US and in European countries [26, 27].

The most common PIP in the present study as well as in other enquiry was antidepressants, anxiolytics and antipsychotics [6]. STOPP criteria revealed higher prevalence of PIP than Beers criteria. Previous researches also have shown differences between the two criteria [28, 29].

The difference found in this research can be attributed to lack of detailed information in the medical records, e.g. there were not records about kidney function, history of falls, syncope or delirium in the medical records. This may have influenced in the lower prevalence of PIP when Beers criteria were applied. Some antidepressants, such as the benzodiazepines and antipsychotics prescribed should be avoided in elderly people with a history of falls and fractures, but this information was not available in the records.

Besides, the criteria developed in Europe (STOPP) and in the US (Beers) might not coincide with the Brazilian real scenario, considering the essential medicines list used in the different settings. Further research is needed to confirm this hypothesis.

While the Brazilian records show this scenario of high consumption of several classes of inappropriate medications (long-acting benzodiazepines, tricyclic antidepressants, antipsychotics), in many other countries, physicians have moved away from these agents. Several countries are actively encouraged to reduce the use of psychiatric medications when risks outweigh benefits, mainly for tricyclic antidepressants, antipsychotics and fall-risk medicines. Such attempts have been successful in Canada, in Europe and in the UK [14, 17, 30].

The scenario found in Porto Feliz can be attributed to lack of national criteria, lack of medication monitoring and medication reviews, to poor training of physicians and absence of continued education for the healthcare team. Besides, in countries like Brazil, which have a public health system of universal coverage, it is common to find bad practices by the misuse of medicines. We also can consider the cost of the new antidepressant SSRIs and atypicals. The budget to acquire SSRIs and atypical antidepressants could be insufficient and the physician is led to prescribe old drugs (tricyclic antidepressants) now regarded as unsafe, if compared with the former.

On the other hand, this may reflect the fact that Beers “considering diagnoses and conditions” criteria accounted for lower PIP prevalence rate than the PIP listed in Beer’s “independent of diagnoses or conditions” criteria.

This study found that elderly female were more exposed to PIM than males, which is similar to the results found by other researchers [31, 32].Our study, as well as other enquiries, reported that patients younger than 75 years of age were more exposed to PIP [26, 33].

Another relevant finding was related to median number of concurrent medicines, which was 3 (interquartile 2–4), a lower number of medicines than in other studies [27, 33]. Patients younger than 75 years of age, with a lower number of comorbidities reflect the lower mean number of medications used and could explain the discrepancy.

In agreement with previous studies in the US and Europe, the use of multiple medications in patients with depression and anxiety were associated with PIP [27, 34].

Tricyclic antidepressants and SSRIs are extensively used, but little is known about risks and serious adverse effects in older people [9]. Approximately 55% of the elderly were taking SSRIs, which agrees with data from other studies [9, 27, 35]. The review suggests that SSRIs and tricyclic antidepressants hold the same efficacy, but they have different side effect profiles and tricyclic antidepressants are associated with a higher withdrawal rate due to side effect experience [4].

Beers classify all tricyclic antidepressants as inappropriate in patients with delirium, history of falls, fractures and syncope due to a highly anticholinergic profile, sedating and orthostatic hypotension. However, this criteria recommends to use SSRIs with caution [8]. STOPP criteria do not mention anything about it.

Cohort study conducted in United Kingdom found that SSRIs were associated with the highest adjusted hazard ratios for falls and hyponatremia comparing when antidepressants were not being used [9].

The benzodiazepines are the most common medications prescribed as anxiolytic or as sleep aid among elderly patients [35].Old patients who are sick are more likely to use benzodiazepines inappropriately in the long-term, without evidence for the effectiveness in chronic use [36]. Long-term use (more than 30 days) is contraindicated in elderly and considered as potentially inappropriate medication in STOPP and Beers criteria due to the risk of prolonged sedation, confusion, psychomotor impairment, falls and physical dependence [5, 7, 25].

Benzodiazepines and antipsychotics are the most potentially inappropriate medications for elderly people, given their adverse effects. The findings of this study are consistent with those from the US and European countries, in which most patients also used this medication for a long term combined with other psychotropic and/or used duplicate drugs (mainly antidepressants and anxiolytic/ hypnotics) [26, 27]. STOPP recommends not using antipsychotics for more than 1 month due to risk of confusion, hypotension, extrapyramidal side effects and falls [7].

The impact of safety warnings on the use of antipsychotic drugs has been assessed in some countries to protect vulnerable populations from potentially hazardous medications and it seems that the use of first generation antipsychotics has declined over the time [15, 16].

### Limitations and strengths

These findings represented important Brazilian data on the use of psychotropic drugs in elderly outpatients taking antidepressants from public health services. We obtained pharmacy records of medication use and corroborating information from medical records and then cross-checked information to ensure accuracy.

In addition, the findings represent valuable information about drug therapy for the elderly, mainly among psychiatrists and physicians in general. This is because they provide a true picture of clinical practice in the setting of the public health system in Brazil.

There are limitations in this study. We were unable to obtain relevant data from a substantial number of patients, because most of the medical records did not have detailed information, which may underestimate the data. Our estimates may represent only a fraction of inappropriate psychotropic drug use in elderly patients. Besides, we did not investigate the effect on patient outcome.

## Conclusions

The present study showed that regardless of the criteria applied, the prevalence of PIP in elderly patients assisted by the Brazilian Public Health Service was high. Electronic implementation of criteria (STOPP or Beers) in the public health services would represent an important tool for clinicians to guide prescription to the elderly, to improve the quality of the use of psychotropic and to decrease adverse outcomes and costs.

Different initiatives by Brazilian government could contribute to decrease the inappropriate prescription to elderly patients. Safety warning, guidance, increased public health surveillance of psychiatric medication, regular and comprehensive medicine reviews, improved recognition of fall-risk medicines by all clinicians, support to prescribers by implementing national criteria for deprescribing, follow-up and monitoring are some examples used by other countries.

Given the ageing of the population and the risks of PIP, it is necessary to develop and validate easily applicable, evidence-based national criteria and to apply them in an effective way, according to Brazilian scenario.

“Less is more” as regards the elderly; therefore, safer pharmacological alternatives as well as non-pharmacological ones could be available to pose more benefits than risks.

## Abbreviations

ATC: Anatomical therapeutic chemical system
CNS: drugs - central nervous system active drugs
HDI: Human development index
ICD-10: International classification of disease version 10
PIM: potentially inappropriate medication
PIP: potentially inappropriate psychotropic
SSRIs: Selective serotonin reuptake inhibitor antidepressants
STOPP: Screening tool in older persons’ prescriptions
TCAs: tricyclic antidepressants
